# Linking Functional Traits to Anthropogenic Stress: A Dung Beetle Case Study in a Degraded Habitat

**DOI:** 10.1007/s13744-026-01414-6

**Published:** 2026-08-13

**Authors:** Bruna Raiary, Hernani Alves Almeida, Wallace Beiroz, Yasmine Antonini

**Affiliations:** 1 Programa de Pos Graduação Em Ecologia de Biomas Tropicais-UFOP, Ouro Preto, Minas Gerais Brazil; 2 Programa de Pos Graduação Em Ecologia E Sustentabilidade, Departamento de Engenharia E Meio Ambiente-UFPB, João Pessoa, Paraíba Brazil; 3 Instituto Nacional de Coleoptera (INCol), Instituto Nacional de Ciência e Tecnologia (INCT) – CNPq, Brasília, Brazil

**Keywords:** Functional Diversity, Secondary Seed Dispersal, Scarabaeinae, Ecological Restoration, Bioindicators, Functional Traits, Cerrado

## Abstract

Anthropogenic disturbances, such as extensive topsoil removal, pose severe threats to ecosystem integrity, and consequently restoration outcomes depend heavily on biotic interactions that sustain ecosystem functions. Dung beetles (Coleoptera: Scarabaeinae) act as bioindicators and ecosystem engineers, notably by mediating secondary seed dispersal and promoting soil bioturbation. We investigated taxonomic and functional attributes of dung beetle communities and quantified seed-removal rates in a severely degraded site (topsoil removal for dam construction) and in an adjacent, less-disturbed reference area within the Brazilian Cerrado. Contrary to our initial predictions, in the degraded site hosted higher species richness (27 vs. 26 species), greater abundance (5371 vs. 1142), and higher functional richness (FRic), whereas functional divergence (FDiv) was greater in the reference area. Community-weighted means (CWMs) for traits associated with excavation and rolling (pronotum volume, front-leg area, and hind-to-front leg length ratio) were significantly elevated in the degraded area, despite a lower mean body mass. Seed-removal rates were also significantly higher in the degraded site. Across the dataset, seed removal correlated positively with FRic, abundance, pronotum volume, front-leg area, and hind/front leg ratio, and negatively with FDiv and body mass; however, these relationships were context-dependent and largely vanished when habitats were analysed separately. Our results indicate that the dung beetle assemblage colonizing the degraded site is dominated by abundant, morphologically specialized species that can sustain high rates of seed removal despite low functional divergence. We discuss implications for restoration: the presence of functionally competent, abundant dung beetles can facilitate secondary seed dispersal in heavily degraded landscapes, but reliance on a few trait-dominant species may render this service vulnerable to further perturbation.

**Supplementary Information:**

The online version contains supplementary material available at 10.1007/s13744-026-01414-6.

## Introduction

Environmental degradation is a major challenge for conservation in the Brazilian Cerrado, resulting in biodiversity loss and impairment of critical ecosystem functions (Schwaida et al. 2023; Machado et al. 2024; Pilon et al. 2025). Conversion of natural habitats, suppression of native vegetation and removal of surface soil layers instigate severe degradation processes—exposure and compaction of soils, loss of organic matter, habitat loss, altered biotic interactions and reduced ecosystem resilience (Chazdon 2008; Antonini et al. 2021). Thus, restoration strategies should extend beyond vegetation reestablishment to include the maintenance of organisms that provide key ecosystem services, thereby accelerating recovery and reducing restoration costs (Ferreira et al. 2007).

Dung beetles (Coleoptera: Scarabaeinae) are prominent secondary seed dispersers and are widely recognised as bioindicators of habitat quality (Nichols et al. 2008). By removing and burying feces they reduce seed predation, enhance germination conditions and transport seeds to microsites favourable for establishment, catalysing natural regeneration in degraded landscapes (Andresen & Levey 2004; Andresen & Feer 2005; Gandolfi and Rodrigues 2007). Here we focus on functional effect trait analyses, particularly morphological attributes such as body mass, pronotum volume and leg morphology potentially affecting seed-removal capacity, which allows to understand the mechanism linking species' traits to their contributions to ecosystem processes (Decastro-Arrazola et al. 2020; Giménez Gómez et al. 2022).

Functional diversity is increasingly used to relate trait variation to ecosystem service provision (Mouchet et al. 2010; Mouillot et al. 2013). Empirical and theoretical work suggests that functionally diverse communities can sustain more stable and efficient ecosystem functioning (Griffiths et al. 2015). However, disturbed habitats often lose large-bodied mammals, and this can reduce dung beetle’s specialist on feces of these animals. This loss can affect disproportionately functional diversity, leading to reduced taxonomic and functional diversity (Larsen et al. 2008; Nichols et al. 2009). In dung beetles, traits related to excavation (e.g. pronotum robustness and front-leg morphology) and to rolling (e.g. hind-leg length relative to front-leg length) are directly linked to seed dispersal potential (Pessôa et al. 2017; Bui et al. 2020), and selective loss of such traits could impair functional performance in degraded sites (Flynn et al. 2009; Alvarado et al. 2019), unless if compensatory morphotypes persist (Petchey & Gaston 2006).

Conversely, communities with high functional divergence may exploit resources more complementarily, increasing ecosystem efficiency (Mason et al. 2005; Cadotte et al. 2011; Derhé et al. 2016), suggesting positive associations between functional diversity and seed-removal rates. Because relatively intact habitats often support more abundant and functionally capable communities, we initially predicted higher seed-removal rates in the reference area than in the degraded area (Slade et al. 2007; Nichols et al. 2008).

Here we examined how dung beetle’s effect traits changes with severe degradation caused by vegetation suppression and extensive topsoil removal, and whether such communities retain the capacity to provide secondary seed-dispersal. Specifically, we compared taxonomic and functional attributes of dung beetle assemblages and measured seed-removal rates in a degraded site and an adjacent reference area in the Cerrado. We tested four hypotheses: (i) Severe degradation reduces dung-beetle abundance and richness, resulting in a narrower range and less even distribution of effect traits, expressed as lower FRic, FDis, FDiv, and FEve. (ii) Species dominating degraded areas are smaller-bodied and exhibit proportionally reduced pronotum and legs, indicating morphological constraints that limit excavation and rolling efficiency. (iii) Seed removal increases with the abundance and dominance of morphologically efficient species rather than with overall functional diversity. (iv) Seed removal is higher in the reference area.

## Materials and Methods

### Study Area

**Fig. 1:**
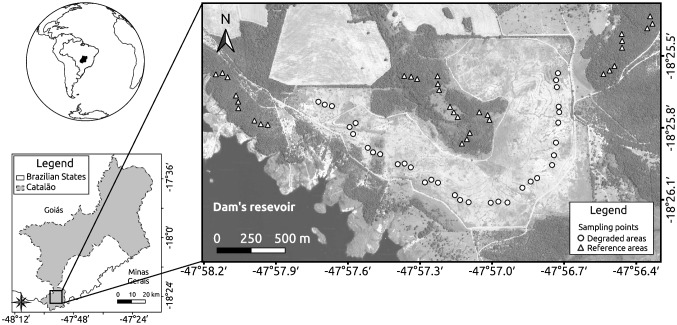
Location of the study area and sampling points in the reference (triangles) and degraded (circles) sites

Sampling was conducted in January 2019 at a site affected by topsoil removal for the construction of the Emborcação Hydroelectric Dam, near Catalão, Goiás, Brazil (18° 26′ 00″ S, 47°56′57.41" W; Fig. 1). The region lies in a transition between Cerrado and Atlantic Forest physiognomies and has a Köppen Aw climate: a wet season from October to March and a dry season from April to September (Cardoso 2014). Mean annual precipitation is ~ 1,500 mm and mean temperature for 2000–2018 was ~ 23 °C (Almeida et al. 2022).

We sampled two habitat types: a reference area and a degraded area. The reference area consisted of dense Cerrado fragments totaling approximately 98 ha, subject to sporadic anthropogenic use such as grazing but retaining native vegetation (Mastella et al. 2019). Although no pristine sites were available, the selected reference fragment retains native vegetation and soil structure representative of regional Cerrado physiognomies. Adjacent fragments with distinct disturbance histories were chosen to maximise environmental comparability while isolating the effect of topsoil removal.

The degraded area extends over ~ 190 ha and was affected by removal of ~ 5 m of topsoil during construction of the Emborcação dam around the 1980 s (Ziade et al. 2022). Despite post-removal recovery attempts, restoration was largely unsuccessful and ~ 65% of the degraded surface remains exposed (Ziade et al. 2022). Sampling was conducted within a single degraded patch where topsoil removal occurred uniformly. Individual transects were treated as independent sampling units, providing spatially replicated measures of beetle and functional diversity within the disturbance type.

### Dung Beetle Sampling

We collected dung beetles using pitfall traps. Each trap consisted of a plastic container (15 cm diameter × 9 cm depth) buried flush with the soil surface and baited with a 1:1 mixture of human and pig feces (Marsh et al. 2013). The sampling design included 11 transects per habitat (degraded and reference), each 100 m in length and separated by at least 150 m (Silva & Hernández 2015; Fig. 1). Three traps were installed per transect, spaced 50 m apart (Almeida et al. 2022). Traps were active for 48 h and baits were refreshed every 24 h to maintain comparable attractiveness across habitats. To reduce rain inundation and desiccation, each trap was covered by a lid suspended ~ 10 cm above the opening.

Specimens were preserved in 70% ethanol in the field and later oven-dried at 60 °C for 48 h to constant weight. Taxonomic identification was made to species or morphospecies using specialised keys and comparison with reference material from the Scarabaeidology Laboratory collection at the Federal University of Mato Grosso (Vaz-de-Mello et al. 2011).

### Functional Trait Measurement and Functional Diversity Indices

Captured specimens were prepared for morpho-functional analyses. Linear measurements (length and area) were taken using a Zeiss stereomicroscope coupled to Zeiss Zen software. Body mass was measured with a SHIMADZU analytical balance (model AY220) to a precision of 0.0001 g. We selected functional traits that approximate secondary seed-dispersal potential via excavation and rolling behaviours (see Griffiths et al. 2015): pronotum volume (linked to excavation force because prothoracic muscles attach in this region), front-leg length (primary structure for digging and manipulating resources), hind-leg length (important for rolling behaviour and propulsion), front-leg area (complementary to digging capacity), and body mass (used as a proxy for physical strength, resource use and dispersal capacity; Pessôa et al. 2017).

Functional indices were calculated using four traits: (i) pronotum volume and (ii) front-leg area, both divided by body mass to reduce body-size bias; (iii) the ratio of hind-leg to front-leg length; and (iv) body mass as a separate trait. Using these traits we computed Functional Richness (FRic), Functional Evenness (FEve), Functional Divergence (FDiv) and Functional Dispersion (FDis) with the R package dbFD (Laliberté & Legendre 2010; R Core Team 2025). Each metric captures a distinct aspect of community functional structure: FRic quantifies the volume of trait space occupied; FEve measures regularity of species' abundance distribution across trait space; FDiv quantifies whether abundance is concentrated at trait-space extremes; and FDis is the abundance-weighted mean distance of species to the functional centroid (Villéger et al. 2008; Laliberté & Legendre 2010).

We also computed community-weighted means (CWMs) for each trait to characterise the dominant trait values in each area, alongside species richness and abundance per area. CWMs were calculated using species’ relative abundances as weights, so that the resulting values represent the average trait configuration of the individuals active in each transect (Lavorel and Garnier 2002).

### Seed Removal Experiment

Seeds of *Solanum lycocarpum* A. St.-Hil. (Solanaceae; “lobeira”) were extracted from ripe fruits and stored in paper bags for use in seed-removal assays (Almeida et al. 2022). This pioneer species is native to the region and constitutes a key food resource for vertebrates and invertebrates (Rizzini 1971; Vidal et al. 1999; Mota Júnior & Martins 2002).

One experimental arena was established at the central point of each transect (11 arenas total). Each arena was circular with 4 m diameter and bounded by a 2.6 mm^2^ mesh that constrained lateral beetle movement while leaving the top open to allow free access by attracted individuals. Leaf-litter was removed from the interior of arenas to facilitate observation. At the centre of each arena we placed 300 g of bait (1:1 mix of human and pig feces) into which 200 lobeira seeds were incorporated. Baits remained in the field for 24 h. Seed-removal rate was calculated as (Ni – Nf)/Ni, where Ni is initial seed number and Nf is the number of seeds recovered from the fecal mass after the exposure period. We assumed that seeds not recovered at the deposition point had been dispersed or buried by beetles (Almeida et al. 2022). Seed removal experiment was done before the sampling of beetles to avoid interference on the baits.

### Data Analysis

All analyses were conducted at the transect level (n = 11 per habitat) to avoid pseudoreplication. We tested data normality using Shapiro–Wilk; given small sample sizes (< 30) and the limited utility of this test for small datasets, we also inspected histograms and QQ-plots. As none of the response variables (species richness, abundance, FRic, FEve, FDiv, FDis, CWMs, seed-removal rates) met normality assumptions, we used non-parametric Mann–Whitney tests with habitat (degraded vs. reference) as the grouping factor. Relationships between diversity metrics and seed-removal rates were assessed with Spearman rank correlations.

Because FRic can be inflated by rare species, we recalculated it after excluding singletons and doubletons. We then assessed linear correspondence between the original and recalculated values using Pearson’s correlation and compared habitats with Mann–Whitney tests to verify whether the exclusion of rare species affected between-habitat differences.

## Results

**Fig. 2:**
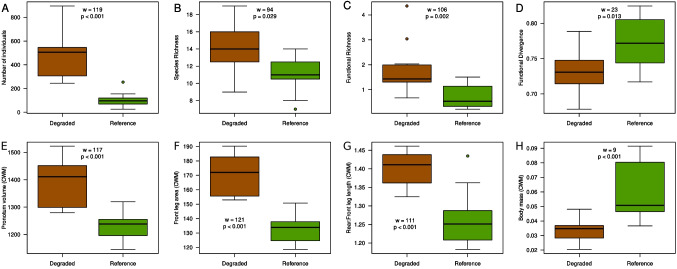
Comparison between degraded and reference areas for dung beetles (Scarabaeinae). (**A**) Individual abundance; (**B**) Species richness; (**C**) Functional richness (FRic); (**D**) Functional divergence (FDiv); (**E**) Pronotum volume (CWM); (**F**) Front-leg area (CWM); (**G**) Ratio between hind and front legs (CWM); (**H**) Body mass (CWM). Communities in degraded areas showed higher abundance, species richness, functional richness, pronotum volume, front-leg area, and hind/front leg ratio, whereas body mass and functional divergence were higher in the reference areas

We sampled 6513 individuals representing 34 dung-beetle species. The degraded area supported a more abundant and species-rich community (Fig. 2A–B): 5371 individuals of 27 species were recorded in the degraded area, compared with 1142 individuals of 26 species in the reference area. Species composition differed substantially between habitats: eight species were found exclusively in the reference area and 8 exclusively in the degraded area (Supplementary Material 1).

To measure functional traits to calculate the functional diversity indices we used a minimal of five and a maximum of 30 individuals of 36 species, totaling 711individuals Contrary to initial expectations, functional richness (FRic) was higher in the degraded area, while functional divergence (FDiv) was greater in the reference area (Fig. 2C–D). Functional evenness (FEve; W = 58; p = 0.898) and functional dispersion (FDis; W = 43; p = 0.270) showed no differences between habitats, indicating comparable abundance distribution and trait-space dispersion.

To ensure that FRic pattern was not driven by rare species, we tested the sensitivity of FRic to singletons and doubletons. Excluding these taxa slightly reduced the quality of the functional space representation (≈0.76 after removal), but recalculated values remained moderately correlated with the original estimates, whether singletons (r = 0.467, p = 0.028) or doubletons (r = 0.634, p = 0.001) were excluded. Differences between habitats also remained significant in all cases (W = 94, p = 0.030; W = 106, p = 0.003, respectively), indicating that the observed functional structure was robust to the influence of rare species.

Morphological traits also varied between habitats. Beetles in the degraded area had larger pronotum volume, front-leg area, and hind/front-leg ratio, whereas body mass was greater in the reference area (Fig. 2E–H).

**Fig. 3:**
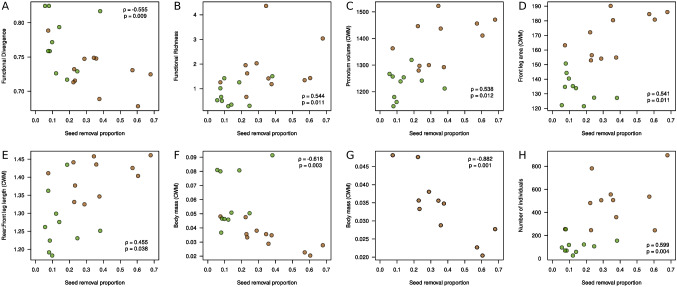
Spearman correlations between seed-removal rate and functional diversity metrics and community-weighted means. Points are coloured by habitat (orange = degraded, green = reference). Only significant correlations (p < 0.05) are shown. ρ and p-values refer to the pooled analysis

When data were pooled across habitats, seed-removal rate increased with FRic, abundance, and CWMs of pronotum volume, front-leg area, and hind/front-leg ratio, but decreased with FDiv and body mass (Fig. 3). FEve, FDis and species richness were not significantly correlated with seed removal (p ≥ 0.05). When analyses were split by habitat, most correlations were no longer significant (p ≥ 0.05), except for a strong negative correlation between body mass and seed removal in the degraded area (Fig. 3G).

**Fig. 4:**
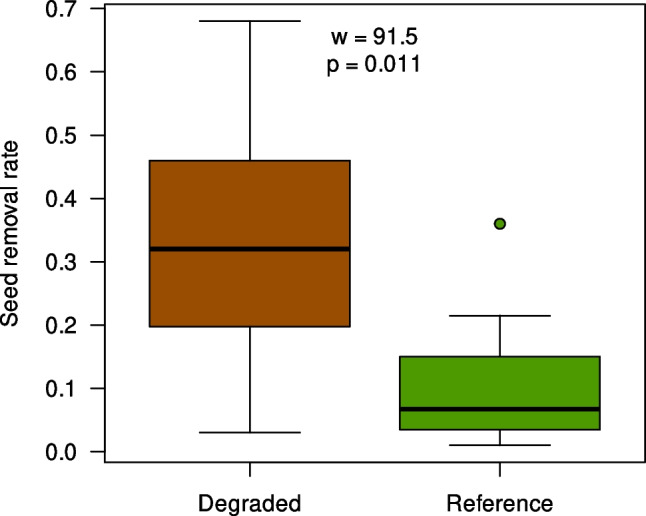
Seed-removal rate by dung beetles in degraded and reference areas

Seed-removal rates were higher in the degraded area (Fig. 4), suggesting that despite taxonomic and morphological shifts, functional performance was maintained, or even enhanced, under severe degradation, consistent with the trait-reorganization hypothesis.

## Discussion

### Taxonomic and Functional Aspects of the Communities

Our findings reveal a complex response of dung-beetle communities to severe habitat degradation. Contrary to initial expectations, the degraded area exhibited greater species richness and abundance. This pattern likely reflects a community dominated by species tolerant of open, simplified habitats (Bitencourt et al. 2019; Silva et al. 2014). Increased availability of faecal resources—perhaps due to aggregation of generalist mammals in open areas—may attract dung beetles in the degraded area, although we cannot rule out that cross-habitat movement from nearby Cerrado remnants also contributes to the observed richness and abundance. However, these taxonomic metrics represent community response to disturbance rather than their functional effect; high richness does not necessarily imply higher ecological performance (Lavorel and Garnier 2002).

Functional structure also responded strongly to degradation. The greater FRic in the degraded area indicates that the range of morphological traits associated with seed removal expanded under disturbance, reflecting the proliferation of species capable of excavation and rolling (Milotić et al. 2018), besides in this area we found higher abundance of small beetles, which are less efficient at removing excrement than larger species.

In contrast, the lower FDiv in the degraded area indicates that abundant species are clustered near the functional centroid, implying low functional complementarity and potentially increased competition for similar resources (Mason et al. 2005; Villéger et al. 2008). The combination of expanded trait range and low trait divergence, in tandem with high abundance, suggests that ecological function can persist through dominance of morphologically similar but effective taxa, a signature of effect-trait redundancy rather than diversity-driven complementarity (Petchey & Gaston 2006; Rocha-Santos et al. 2020). By contrast, the reference area exhibited higher FDiv, although supporting fewer species, suggesting a community with more functionally divergent and complementary species.

The elevated FRic observed in the degraded site likely reflects genuine differences in trait-space volume between habitats, as sensitivity analyses indicate that the functional structure is robust to the exclusion of rare species; however, caution is warranted, as the potential for rare species to inflate FRic may be context-dependent and should be considered in other studies (Umaña et al. 2017; Chapman et al. 2018).

Community-weighted mean traits reveal how morphological efficiency compensates for body mass reduction in degraded site. Larger mean body mass in the reference area aligns with numerous studies reporting loss of large-bodied dung beetles under anthropogenic disturbance (Scheffler 2005; Larsen et al. 2008). Large paracoprid species are effective in removing large quantities of dung and are favoured in stable habitats (Nervo et al. 2014; Bui et al. 2020). Conversely, the degraded area was dominated by smaller-bodied beetles—a phenomenon frequently reported in disturbed habitats and consistent with resilience strategies that entail lower resource requirements (Vulinec 2002)—but these individuals displayed relatively larger pronotum, front-leg areas and hind/front leg ratios, traits associated with efficient excavation and rolling, which may compensate for reduced individual size and help maintain ecological function. These configurations likely increase mechanical efficiency per biomass unit, enhancing excavation and rolling performance despite reduced body mass (Marín-Armijos et al. 2023).

Therefore, despite lower body mass, the morphological specialization of species in the degraded area likely underpins the observed high seed-removal rates. The degraded assemblage is characterized by abundant, morphologically specialized species occupying similar trait space (low FDiv) but collectively performing the seed-removal function effectively. This dominance of morphologically specialized, functionally similar species demonstrates a form of functional compensation, where ecosystem function is maintained not through diversity of effect traits, but through redundancy of highly efficient trait expressions (Tonelli et al. 2020). Such redundancy can stabilize process rates under extreme disturbance, exemplifying a pathway of functional resilience (De Bello et al. 2021; Decastro-Arrazola et al. 2023).

### Implications for the Restoration of Degraded Areas

Correlations between trait metrics and seed removal were context-dependent: significant associations observed across the pooled dataset largely disappeared when habitats were analysed separately. This indicates that trait–function relationships are strongly mediated by environmental context (Griffiths et al. 2015). From a restoration perspective, dung beetles can contribute substantially to natural regeneration in degraded areas, but their effectiveness depends on interactions with habitat structure and resource availability rather than on trait distribution alone. Protecting adjacent source habitats is therefore essential to ensure recolonisation and functional stability following stochastic events. We hypothesise that increased abundance of *S. lycocarpum* in the degraded area may enhance seed availability via vertebrate frugivores (e.g. *Lycalopex vetulus*) and indirectly promote dung-beetle activity, although we did not measure vertebrate-mediated seed input directly.

Our results underscore that higher functional divergence does not necessarily translate into higher intensity of ecosystem function; instead, seed-removal services by dung beetles appear to be more tightly linked to the abundance of individuals possessing key morphological traits than to aggregate measures of functional diversity. Conservation and management should therefore emphasise maintaining populations of functionally important trait groups, while also preserving interaction networks, functionally redundant species and source populations to buffer against demographic fluctuations (Beiroz et al. 2019; Nunes et al. 2021).

## Supplementary Information

Below is the link to the electronic supplementary material.

ESM 1 (DOCX 147 KB)

## Data Availability

The data set will be provided under request.
