# Supplementary material for: Linking Functional Traits to Anthropogenic Stress: A Dung Beetle Case Study in a Degraded Habitat
Source: Neotrop Entomol. 2026 Aug 13;55(1):75. doi: 10.1007/s13744-026-01414-6 (PMC13473256; doi:10.1007/s13744-026-01414-6)
Supplement: Supplementary file 1 [file 13744_2026_1414_MOESM1_ESM.docx]

Table 1. Abundance of species of dung beetles sampled in the Degraded and Reference areas.

| **Beetle specie** | **Degraded** | **Reference** | **Total** |
| --- | --- | --- | --- |
| *Agamopus viridis* Boucomont, 1928 | 3 | 0 | 3 |
| *Ateuchus striatulus* (Preudhomme de Borre, 1886) | 1 | 1 | 2 |
| *Canthidium* aff. *barbacenicum* Preudhomme de Borre, 1886 | 11 | 0 | 11 |
| *Canthidium  barbacenicum* Preudhomme de Borre, 1886 | 29 | 29 | 58 |
| *Canthidium impressum* Boucomont, 1928 | 2 | 0 | 2 |
| *Canthidium* sp. | 0 | 30 | 30 |
| *Canthon* aff *simulans* (Martínez, 1950) | 7 | 18 | 25 |
| *Canthon conformis* Harold, 1868 | 1 | 31 | 32 |
| *Canthon  histrio* Lepeletier de Saint Fargeau & Audinet-Serville, 1828 | 30 | 28 | 58 |
| *Canthon* sp. | 4 | 0 | 4 |
| *Canthon  virens* (Mannerheim, 1829) | 8 | 0 | 8 |
| *Coprophanaeus cyanescens* (Olsoufieff, 1924) | 0 | 1 | 1 |
| *Delthorhinum* sp. | 0 | 1 | 1 |
| *Deltochilum* aff. *komareki* Balthasar, 1939 | 10 | 14 | 24 |
| *Deltochilum enceladus* Kolbe, 1893 | 0 | 15 | 15 |
| *Deltochilum  pseudoicarus* Balthasar, 1939 | 8 | 0 | 8 |
| *Dichotomius* aff. *carbonarius* (Mannerheim, 1829) | 5 | 30 | 35 |
| *Dichotomius* aff. *cuprinus* (Felsche, 1901) | 0 | 18 | 18 |
| *Dichotomius* aff. *glaucus* (Harold, 1869) | 1 | 0 | 1 |
| *Dichotomius* aff. *lycas* (Felsche, 1901) | 1 | 0 | 1 |
| *Dichotomius bicuspis* (Germar, 1824) | 0 | 1 | 1 |
| *Dichotomius bos* (Blanchard, 1846) | 30 | 15 | 45 |
| *Dichotomius  nisus* (Olivier, 1789) | 30 | 30 | 60 |
| *Dichotomius* *reichei* (Germar, 1824) | 0 | 5 | 5 |
| *Dichotomius zikani* (Luederwaldt, 1922) | 0 | 2 | 2 |
| *Digitonthophagus gazella* (Fabricius, 1787) | 1 | 0 | 1 |
| *Genieridium bidens* (Balthasar, 1938) | 7 | 0 | 7 |
| *Ontherus appendiculatus* (Mannerheim, 1829) | 15 | 0 | 15 |
| *Ontherus* sp. | 4 | 2 | 6 |
| *Onthophagus buculus* Mannerheim, 1829 | 29 | 31 | 60 |
| *Onthophagus ptox* Erichson, 1847 | 29 | 0 | 29 |
| *Oxysternon palaemon* Castelnau, 1840 | 27 | 0 | 27 |
| *Pseudocanthon* sp. | 26 | 0 | 26 |
| *Tetraechma liturata (*Germar, 1822) | 28 | 0 | 28 |
| *Trichillum externepunctatum* Preudhomme de Borre, 1880 | 27 | 0 | 27 |
| *Uroxys* aff. *thoracalis* Balthasar, 1940 | 4 | 31 | 35 |

***Table 2.*** Results of the calculation of the four functional diversity indices based on functional traits. FRic: Functional richness; FEve: Functional evenness; FDiv: Functional divergence; FDis: Functional dispersion.

| **Cod** | **Area** | **FRic** | **FEve** | **FDiv** | **FDis** |
| --- | --- | --- | --- | --- | --- |
| D1 | Degraded | 1.6210652 | 0.7468587 | 0.7156159 | 1.284289 |
| D2 | Degraded | 2.0300141 | 0.5763110 | 0.7473736 | 1.425777 |
| D3 | Degraded | 1.1847030 | 0.5804240 | 0.6888209 | 1.308115 |
| D4 | Degraded | 0.6659714 | 0.8005958 | 0.7322519 | 1.245059 |
| D5 | Degraded | 1.2592325 | 0.7042459 | 0.7885131 | 1.377505 |
| D6 | Degraded | 1.3449780 | 0.7729458 | 0.7308430 | 1.267358 |
| D7 | Degraded | 1.4165126 | 0.7830473 | 0.7477904 | 1.276785 |
| D8 | Degraded | 3.0388358 | 0.7021157 | 0.7247285 | 1.320498 |
| D9 | Degraded | 1.4297330 | 0.4579559 | 0.6782810 | 1.517673 |
| D10 | Degraded | 1.9534432 | 0.6093997 | 0.7131945 | 1.352516 |
| D11 | Degraded | 4.3597133 | 0.6332352 | 0.7488677 | 1.416731 |
| R1 | Reference | 0.3053120 | 0.6935831 | 0.7290851 | 1.225149 |
| R2 | Reference | 0.5060190 | 0.7419147 | 0.7586089 | 1.406372 |
| R3 | Reference | 0.6671011 | 0.7445073 | 0.8245340 | 1.442163 |
| R4 | Reference | 1.0167566 | 0.7031279 | 0.7587081 | 1.682341 |
| R5 | Reference | 0.2889020 | 0.7603113 | 0.7261938 | 1.172682 |
| R6 | Reference | 0.2154510 | 0.6244495 | 0.7827348 | 1.462411 |
| R7 | Reference | 0.3529779 | 0.7070441 | 0.7936472 | 1.262287 |
| R8 | Reference | 1.4226632 | 0.5855523 | 0.7716801 | 1.442324 |
| R9 | Reference | 1.2587806 | 0.6784009 | 0.7169214 | 1.352811 |
| R10 | Reference | 0.5314871 | 0.6173838 | 0.8244786 | 1.441588 |
| R11 | Reference | 1.5056041 | 0.7288023 | 0.8168577 | 1.627083 |

***Table 3. Results of the calculation of the community-weighted mean (CWM) of functional traits.***

| **Cod** | **Prono_Vol** | **Hind_area** | **Hind_Front** | **Body_mass** |
| --- | --- | --- | --- | --- |
| D1 | 1297.369 | 156.4237 | 1.376918 | 0.03329401 |
| D2 | 1300.120 | 154.0895 | 1.324849 | 0.03803664 |
| D3 | 1292.378 | 154.8551 | 1.346552 | 0.03478177 |
| D4 | 1279.675 | 152.9017 | 1.331344 | 0.03562043 |
| D5 | 1362.959 | 163.2211 | 1.411088 | 0.04810277 |
| D6 | 1456.269 | 184.6082 | 1.425523 | 0.02268335 |
| D7 | 1437.340 | 180.4098 | 1.435267 | 0.02878179 |
| D8 | 1470.796 | 185.9875 | 1.460874 | 0.02771570 |
| D9 | 1411.138 | 180.7989 | 1.403631 | 0.02043908 |
| D10 | 1446.539 | 172.0965 | 1.441311 | 0.04760862 |
| D11 | 1522.517 | 190.1918 | 1.457790 | 0.03558345 |
| R1 | 1241.661 | 127.3186 | 1.230720 | 0.05038183 |
| R2 | 1179.971 | 144.2756 | 1.224195 | 0.04660974 |
| R3 | 1145.540 | 150.7262 | 1.191630 | 0.03667565 |
| R4 | 1257.128 | 134.7223 | 1.362364 | 0.08002134 |
| R5 | 1238.724 | 135.2702 | 1.298948 | 0.04586125 |
| R6 | 1220.769 | 118.5934 | 1.184632 | 0.07527264 |
| R7 | 1253.985 | 133.8418 | 1.275894 | 0.05080497 |
| R8 | 1161.243 | 140.4445 | 1.182765 | 0.04634289 |
| R9 | 1319.503 | 121.5002 | 1.434497 | 0.08072537 |
| R10 | 1266.886 | 122.1948 | 1.261878 | 0.08100680 |
| R11 | 1212.045 | 127.1717 | 1.251376 | 0.09141393 |
